# Influence of Patient Anatomy on Intraoperative Radiation Exposure and Operation Time during Standard EVAR

**DOI:** 10.3390/jcm12185851

**Published:** 2023-09-08

**Authors:** Wojciech Derwich, Alexandru Barb, Thomas Vogl, Kyriakos Oikonomou, Daphne Gray

**Affiliations:** 1 Department of Vascular and Endovascular Surgery, University Hospital Frankfurt Goethe University, Theodor-Stern-Kai 7, 60590 Frankfurt am Main, Germany; alexandru.barb@icloud.com (A.B.); kyriakos.oikonomou@kgu.de (K.O.); daphne.gray@kgu.de (D.G.); 2 Institute for Diagnostic and Interventional Radiology, University Hospital Frankfurt Goethe University, Theodor-Stern-Kai 7, 60590 Frankfurt am Main, Germany; thomas.vogl@kgu.de

**Keywords:** EVAR, radiation exposure, anatomy

## Abstract

Endovascular aortic repair (EVAR) is the primary treatment for abdominal aortic aneurysms (AAAs). To optimise patient safety during the standard EVAR procedure, we aimed to investigate the influence of patient anatomy on intraoperative radiation exposure and surgical time. This retrospective study comprised 90 patients (mean age 73.4 ± 8.2 years; 92.2% male) with an infrarenal aortic aneurysm who underwent a standard EVAR procedure. The relationships between dose area product, operating time, and anatomical conditions were investigated in preoperative computed tomography angiography using open-source software. Logistic regression analysis indicated that only body mass index (BMI) had predictive value for radiation exposure. The accuracy of the model was 98.67%, with an area under the curve of 0.72. The duration of surgery was significantly correlated with an increased BMI (odds ratio (OR) = 1.183; *p* < 0.05), the tortuosity of AAAs (OR = 1.124; *p* < 0.05), and the left common iliac artery (OR = 1.028; *p* < 0.05). Thus, BMI impacts the prediction of intraoperative radiation exposure more significantly than the anatomical characteristics of the infrarenal aorta and iliac arteries, and the duration of surgery significantly correlates with both BMI and the tortuosity of the infrarenal aorta and iliac arteries.

## 1. Introduction

Endovascular treatment has become the standard therapy for abdominal aortic aneurysms (AAAs). According to the German Aortic Registry, from 2019, 80% of AAAs are treated with endovascular aortic repair (EVAR), while only 20% are treated with open aortic repair [1]. With increasing experience in vascular centres, EVAR is increasingly being utilised for patients with complex anatomies in the access vessels and the landing zones [2]. However, in these cases, surgical success is associated with high consumption of materials and contrast medium, as well as extended surgical and radiation exposure. Previous studies have focused on the effects of anatomical conditions on perioperative complications and surgical outcomes [2,3]. Speziale et al. demonstrated that a higher complexity of the aneurysm neck is associated with an increased number of secondary interventions due to high-flow endoleaks and stent graft migration [2]. Moreover, Oliveira et al. posited that an aneurysm diameter of >70 mm and a neck diameter of >30 mm are associated with a doubling of cardiovascular mortality [4]. Nevertheless, the impact of patient anatomy on radiation exposure has been examined in only a few studies [5].

During EVAR treatment, patients are exposed to radiation through pre-, intra-, and postoperative imaging. Moreover, vascular surgeons are exposed to occupational radiation throughout their careers. Therefore, radiation protection is important at every stage of the therapeutic process. Furthermore, the effects of cumulative radiation dose should not be underestimated [6].

An insightful surgical plan with a precise assessment of the aortic and iliac anatomy is crucial for the optimal implementation of EVAR. Most studies have discussed the impact of off-label and on-label use of stent grafts on intraoperative and postoperative complications [2,7]. However, recognising anatomical features that are associated with prolonged surgical duration is an important factor in surgical planning, as it could help with coordinating operation days, selecting procedures for vascular training, and avoiding predictable complications caused by difficult anatomy.

Proper planning is pivotal for not only the uncomplicated implantation of EVAR with good long-term results but also for reduced operating times and radiation exposure [8,9]. Commercially available 3D workstations and open-source image processing software, such as OsiriX Lite^®^ and Horos (the Horos Project), are essential tools for preoperative aortic sizing and the clinical decision-making process. Strøm et al. showed that experienced experts using semiautomatic workstations provide higher accuracy of measurements than novices and intermediates [10]. Open-source image processing software offers comparable measurement results for EVAR planning, with moderate to good interrater agreement, when compared with commercially available planning tools [11]. The use of OsiriX Lite^®^ in the preoperative setting was described by Knöps et al. [11]. However, to date, we have not come across any study on Horos.

This study aimed to demonstrate the reliability of Horos for taking standard measurements for EVAR planning. The objective of this research was to identify the patient-specific anatomical factors contributing to extended radiation exposure and operating times in patients with AAAs undergoing standard EVAR. To achieve this, prediction models were built to determine the factors that predispose individuals undergoing EVAR to excessive radiation exposure and surgical times.

## 2. Materials and Methods

This retrospective study examined a cohort of 167 patients who underwent EVAR for infrarenal aortic aneurysms between January 2014 and June 2019. The study excluded patients who did not have computed tomography (CT) images available in the hospital archives or had images with inadequate quality, such as an incomplete image range of the infrarenal aorta up to the inguinal arteries with a layer thickness of >2 mm and a missing arterial phase. This study only included patients with bifurcated grafts (Medtronic Endurant IIs, Minneapolis, MN, USA; Anaconda Vascutek, Terumo, Inchinnan, Scotland; Cook Zenith Flex, Cook Medical, Bloomington, IN, USA), while cases involving aortic tubes, aorto-uni-iliac grafts, iliac-side branches, and fenestrated/branched prostheses as primary operations were excluded from this study.

This study was registered with the local ethics committee and approved in accordance with the General Data Protection Regulation (no. 20-776, 17 June 2020). The epidemiological patient data, intervention-related in-hospital events, and additional intraoperative procedures were collected from the hospital information system. Due to the retrospective nature of this study, informed consent was waived.

All procedures were performed in the operating theatre on a carbon operating table, using a Philips Veradius, a mobile, manually controlled digital C-arm system. All surgeries were performed by a dedicated vascular team, which comprised an experienced surgical nurse, a senior consultant specialising in vascular and endovascular surgery, and a vascular trainee. No interventional radiologist participated in the surgeries. The standard radiation data, such as radiation time (s), dose area product (Gycm^2^), and amount of contrast medium (mL), were recorded. Low-dose fluoroscopy was performed using pulsed beam fluoroscopy. The radiation protection for patients and staff was strictly implemented according to the as low as reasonably achievable principle. The postoperative follow-up was limited to the operation-related hospital stay.

### 2.1. EVAR Planning

**Figure 1:**
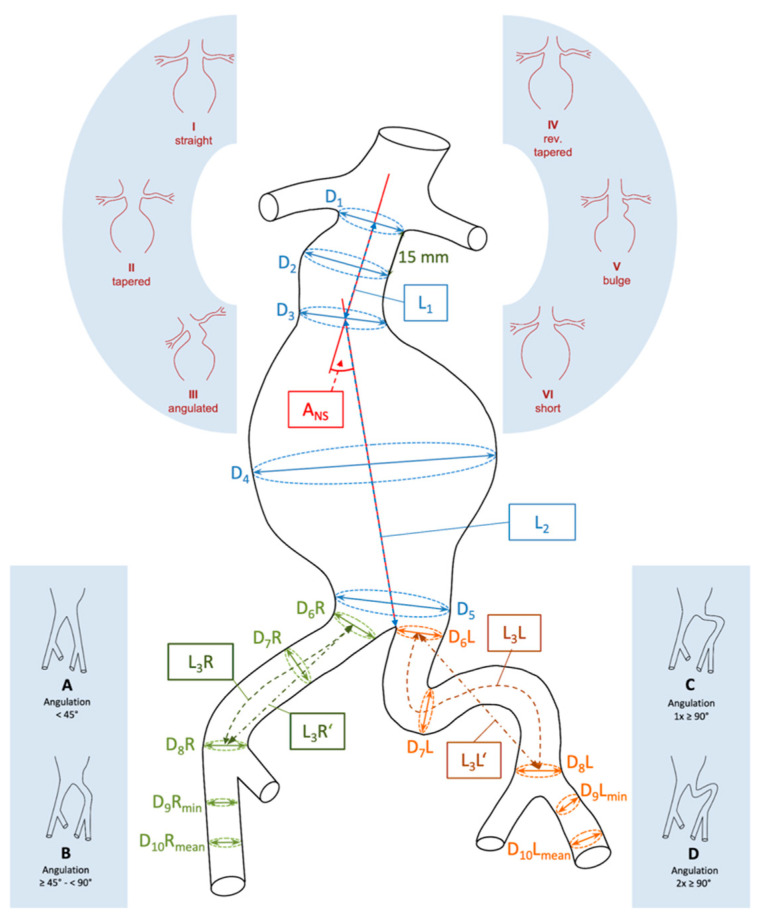
EVAR sizing protocol.

Measurements were performed on the preoperative thin-sliced, arterial-phase CT scans using Horos, a free and open-source medical image viewer for OS X. Horos is available under the GNU Lesser General Public License, Version 3 (LGPL-3.0). The diameters, lengths, and angles of the vessels were measured in the 3D MPR view with manual correction of the centreline, following the standard clinical planning protocol (Figure 1).

The length of the entire infrarenal segment and the common iliac artery was determined using a ruler and a polygon measurement tool. The tortuosity index was calculated separately for the infrarenal aorta and the common iliac artery. It was calculated as the ratio of the vessel length measured along the centreline to the shortest distance between the beginning and end of the segment; this is an example of the tortuosity of the left common iliac artery, denoted as L3L/L3L’. The tortuosity of the common iliac artery was further morphologically evaluated and divided into four grades: Grade I—angulation of <45°; Grade II—angulation of 46–90°; Grade III—one angulation of >90°; and Grade IV—two angulations of >90°. The thrombus and calcifications were assessed in the infrarenal and iliac landing zones, external iliac, and common femoral arteries using a five-grade scale: 0—no thrombus or calcifications; I—<25%; II—26–50%; III—51–75%; and IV—>75% of the vessel circumference. The configuration of the aneurysm neck was characterised as straight, tapered, angulated, reverse tapered, or bulging [12]. We examined the reproducibility of EVAR planning using the Horos viewer in 10 cases. This was performed by measuring the same cases repeatedly by two different observers and calculating the intra- and inter-observer variability.

### 2.2. Statistical Analysis

We analysed all data using the R package ‘stats’, Version 4.1.0 in consultation with the Institute for Biostatistics and Mathematical Modelling at Goethe University Frankfurt. The Kolmogorov–Smirnov test showed a non-normal distribution of the data; therefore, non-parametric tests were used for the statistical analysis. A *p*-value of < 0.05 was considered statistically significant. The influences of independent variables (such as patient-related anatomical characteristics, intraoperative steps, and procedure-related events) on dependent variables (such as radiation exposure and procedure time) were investigated using Spearman’s rank correlation analysis with the determination of the Spearman correlation coefficient (ρ). In the second step, variables with a significant correlation were examined using logistic regression to determine clinically relevant parameters. For this purpose, patients were divided into two groups based on noncritical and critical values for radiation exposure and procedure duration. The cut-off point between patients with noncritical and critical values was set at the mean ± standard deviation (SD). The patients in the critical group had a dose area product of more than 44.03 Gycm^2^ (*n* = 14) and a procedure time of more than 210.7 min (*n* = 9). Logistic regression enables the prediction of the probabilities of belonging to a noncritical or critical group based on a patient’s anatomical characteristics, intraoperative steps, and procedure-related events. Finally, the predictive power of all these parameters for their impacts on radiation exposure and procedure time was stratified by odds ratio (OR) in univariate regression and, if possible, in multivariate regression. The parameters with continuous values were considered significant if the OR was <0.9 or >1.1 and the *p*-value was <0.05. The parameters determined as the quotient of continuous values, such as tortuosity, were considered significant if the OR was <0.99 or >1.01 and the *p*-value was <0.05. The results were graphically displayed as a receiver operating characteristic curve. The predictive value was evaluated based on the area under the curve (AUC). An AUC of >0.9 indicated excellent discrimination, >0.8 indicated good discrimination, >0.7 indicated medium discrimination, and <0.7 indicated poor discrimination [13]. The intra- and inter-observer variability was examined for all measurements by calculating the inter- and intra-class correlation coefficients and interpreting them according to the principles of Landis and Koch [14].

## 3. Results

### 3.1. Patient Characteristics

The hospital data search revealed 167 patients with infrarenal aortic aneurysms treated with EVAR. After the exclusion of 77 patients due to inadequate preoperative imaging, 90 patients (mean age 73.4 ± 8.2 years) were analysed. A total of 83 men (92.2%) and 7 women (7.8%) exhibited a gender-typical age distribution, with men being younger than women (73.8 ± 8.3 vs. 69.4 ± 6.2; *p* < 0.05). However, there were no differences in body mass index (BMI) between the two groups (27.5 ± 4 vs. 27.6 ± 6.9; *p* > 0.05) (Table 1). Due to the unequal gender distribution and low number of females in the cohort, we deliberately omitted gender-based subgroup analysis.

All procedural steps, additional interventions, and complications that might contribute to extended radiation exposure and operating times are summarised in Table 2.

All patients met the criteria outlined in the manufacturer’s instructions for use. All stent grafts, except for three cases, were inserted through the right common femoral artery. No statistically significant difference was observed between the noncritical and critical groups. An aneurysm with a neck angulation above 60° was intended for endovascular treatment only if the neck length exceeded 20 mm. The aneurysm neck had a low thrombus load in 70% of cases (0–25% of the vessel circumference), and only 2.2% of cases (>75% of the vessel circumference) had a high thrombus load. Moreover, the aneurysm neck showed a low calcification grade in 88.9% of cases (0–25% of the vessel circumference). The aneurysm sac had a middle–high thrombus load in 75.6% of cases, which accounted for >50% of the vessel circumference. The distal landing zone in the common iliac artery was characterised by a low thrombus load (0–25% of the circumference), with 82.3% on the right side and 91.1% on the left, as well as a low degree of calcification (0–25% of circumference), with 74.5% on the right side and 67.7% on the left. The external iliac artery on both sides showed a low–moderate calcification grade (<50% of the circumference) in 97.8% of cases. The common femoral artery offered an optimal puncture site with a low calcification grade (0–25% of circumference), with 91.1% on the right side and 90.0% on the left. If present, calcifications were located on the anterior vessel wall in only 2.2% of cases on the right and 4.4% on the left groin. The other anatomical features of the treated aneurysms are described in Table 3.

### 3.2. Intra- and Inter-Observer Variability

The reliability of the measurement results by two observers demonstrates substantial to almost perfect agreement, based on the criteria of Landis and Koch [14] (K > 0.61) (Table 4). Substantial agreement was observed for D3, D5, D6R, D7R, D11L, and InfrarenalB. Moreover, parameters such as A1, D1, D2, D4, D6L, D8RL, D9RL, D10RL, D11RL, L1-L3RL, InfrarenalA, and AICABRL were determined with almost perfect agreement. However, in repeated measurements, the diameter of the common iliac artery showed fair agreement only in the midsection.

### 3.3. Factors Influencing Radiation Exposure

EVAR was associated with acceptable radiation exposure, as measured with the dose area product, with a median value of 14.2 Gycm^2^. However, a broad range of values was observed, ranging from 2.2 to 92.8 Gycm^2^ (Table 5).

Spearman’s rank correlation analysis showed positive correlations between radiation exposure and BMI, the radiation time, the amount of contrast medium, and the type of endograft (Table 6). Radiation exposure was not affected by the proximal landing zone, the maximum aneurysm diameter, the thrombus load, or calcifications. However, the anatomy of the distal landing zone (the diameter of the right common iliac artery) and the vascular access (the diameter of both common femoral arteries and the angulation of the left common iliac artery) seemed to be correlated with a higher dose area product.

The logistic regression revealed that only BMI, and none of the anatomical properties of an AAA, had a predictive value in the prospective assessment of intraprocedural radiation exposure during EVAR (Table 7). The verification of the prediction model for radiation exposure and BMI using K-fold validation showed a high accuracy of 80.1% based on the regression analysis, with a sensitivity of 98.67%. The AUC for BMI was 0.72, indicating good diagnostic quality.

### 3.4. Factors Influencing Procedure Time

Spearman’s correlation test of all the considered parameters indicated a significant influence of both patient-related and anatomical parameters on the intervention time. The BMI and radiation time significantly affected the procedure time. Of the morphological parameters, the neck-sac angulation, maximum aneurysm diameter, tortuosity of the infrarenal aortic aneurysm, tortuosity and angulation of the common iliac artery on both sides (Table 6), and calcification load of the right common femoral artery (ρ = 0.25; *p* < 0.01) significantly correlated with the procedure time. The thrombus load and calcifications in the proximal and distal landing zones, as well as in the other access arteries, did not affect the duration of the operation (ρ < 0.20; *p* > 0.05).

The logistic regression analysis confirmed the clinical significance of BMI (OR = 1.183; *p* < 0.05), the tortuosity of the infrarenal aortic aneurysm (OR = 1.124; *p* < 0.05), and the left iliac common artery (OR = 1.028; *p* < 0.05) on the prolonged duration of the surgery. This study did not observe any effect of the tortuosity of the right common iliac artery on the procedure time (OR = 1.004; *p* > 0.05) (Table 8).

The three parameters mentioned above were used in the prediction model. However, the tortuosity of the left common iliac artery was found to be insignificant (*p* > 0.05) in the Wald test and was therefore excluded from the bivariate analysis. The duration of surgery increased with increasing BMI, with an OR of 1.111 (95% confidence interval (CI): 1.026; 1.218). Additionally, the tortuosity of the infrarenal aortic aneurysm was associated with a higher OR of 1.145 (95% CI: 0.972; 1.355). The K-fold verification of the prediction model for procedure time, based on the BMI and tortuosity of an infrarenal aortic aneurysm, showed an excellent accuracy of 91.8%, with a sensitivity of 98.75%. The AUC values for BMI and the tortuosity of an infrarenal aortic aneurysm were 0.77 and 0.78, respectively. The AUC for both parameters combined was 0.83.

## 4. Discussion

Endovascular treatment for AAAs is considered the gold standard. Anatomical conditions, along with their morphological analysis and non-anatomical, patient-specific parameters, play a central role in the planning of endovascular aneurysm repair [15]. A meta-analysis by Oliveira-Pinto et al. showed in long-term observations a higher rate of endoleak type I in cases of a short neck treated with off-label use of EVAR. Interestingly, severe angulation or a high thrombus load in the proximal neck did not have any effect on the long-term outcome [16]. However, the literature has paid less attention to the effects of patient anatomy on the surgical process. Therefore, the aim of this study was to prospectively model the morphological, patient-specific, and intervention-specific factors that influence the duration of an operation and the amount of radiation exposure during EVAR.

Sobocinski et al. highlighted the advantages of surgical planning using a 3D workstation by utilising multiplanar reconstruction and centreline analysis [8]. Despite the obvious benefits of certified software, the purchase of these types of programmes is associated with significant costs. Knöps et al. confirmed the reliable applicability of open-source software for the adequate planning of EVAR [11]. Our analysis showed almost perfect intra- and inter-observer reliability for all measurements, indicating that the Horos Project software v3.3.6 is a reliable sizing tool regardless of the examiner. However, the basic version of the open-source software offers limited centreline-based measurement and can cause measurement errors for vessel length due to elongated arteries in three-dimensional aspects. Chung et al. mentioned this phenomenon in a 2017 study and associated the relatively low reliability between investigators with the high tortuosity of iliac arteries [17]. Nevertheless, the use of centreline-oriented utility improves the measurement accuracy by less than 2 mm [18]. Thus, the Horos sizing solution is still sufficient for planning simple standard bifurcated stent grafts, but it cannot be used for designing custom-made products in complex anatomical cases.

The adequate protection of patients and hospital staff from X-rays is an essential requirement for modern endovascular surgery. Protective measures include predicting potential conditions that may increase radiation exposure in the future. Our analysis confirms the findings in the literature regarding BMI being a major factor that leads to a significant increase in radiation exposure [19]. Sen et al. showed an approximately two-fold increase in radiation exposure in patients with a BMI of >30 kg/m^2^ compared with patients with a BMI of <30 kg/m^2^ during the implantation of custom-made devices for AAA treatment [20]. In our cohort, where standard bifurcated stent grafts were implanted, we observed that a 1 kg/m^2^ increase in BMI resulted in an 18% increase in the dose area product.

Patient anatomy showed multiple impacts on radiation exposure during EVAR. Calcification and thrombus load in the AAA neck, as well as the length of the aneurysm neck, had no influence on radiation exposure in the study conducted by Machado et al. [5]; this finding is consistent with our study. We could not establish any relationship between the diameter of the aneurysm neck, the angulation between the aneurysm neck and the aneurysm sac, and the maximum aneurysm diameter and the radiation exposure. Machado et al., on the other hand, showed that patients with a neck diameter of >28 mm, neck-sac angulation of >50°, and maximum aneurysm diameter of >60 mm exhibited a higher dose area product than those with lower corresponding values [5]. Badger et al. and Kakkos et al. justified the increased radiation exposure in large aneurysms due to the more challenging cannulation of the contralateral leg of the stent graft and the use of the oblique beam path [21,22]. The current analysis revealed a significant impact of the diameter of the right common iliac artery and the angulation of the left common iliac artery on increased radiation exposure. This finding has not been previously reported in the literature. The validity of the observed indices is questionable because the majority of stent grafts were inserted through the right femoral access. However, achieving accurate visualisation of the distal landing zone in elongated iliac arteries necessitates the use of oblique projections and multiple DSA runs to ensure the precise placement of the stent graft. The elongated iliac arteries complicate wire navigation during the intubation of the contralateral limb and require the establishment of a through-and-through wire, which significantly increases the radiation time. The impact of calcification load in access vessels and significant stenoses on radiation exposure could not be evaluated in this study because most of the patients were excluded from endovascular treatment in favour of open surgery. This justified a low rate of additional interventions and conversions from EVAR to OAR. Although the majority of patients underwent open inguinal surgery, with the percutaneous technique rarely being used, we observed an increase in radiation exposure among patients with a large diameter of the common femoral artery. This observation seems to be related to the ectatic predisposition of the arterial system and the implications described above in the context of the large and elongated iliac arteries. Furthermore, patients with larger femoral arteries also exhibited an increased BMI. Therefore, obesity may serve as a confounding factor and potentially impact the statistical analysis.

Patient anatomy has a greater impact on the duration of the operation than on the level of radiation exposure during EVAR. In this study, the proportion of patients with a BMI of >30 kg/m^2^ in the group with a critical operation time was significantly higher than in the group with a noncritical surgery duration (89% vs. 75%; *p* < 0.05). This confirms the clinical observations that obesity, as indicated by an increased BMI, is significantly correlated with a longer procedure time [19]. This could be attributed to the prolonged duration required for creating inguinal access.

Since all of our patients met the criteria outlined in the manufacturer’s instructions for use, we found no anatomical factors in the proximal landing zone that could have an impact on the duration of surgery. In contrast, Torsello et al. showed a significantly higher intervention time for off-label anatomy, which was described as an aneurysm neck with a length of <15 mm and an angulation of >75° or a neck length of <10 mm and an angulation of <60° [23]. In this study, we found that in cases where an IFU was used, there was a significant association between the prolonged duration of surgery and several factors. These factors included the maximum aortic diameter, the tortuosity of the entire infrarenal aorta, and the angulation with the tortuosity index of the iliac arteries. In perspective, two factors, namely, BMI and the tortuosity of the infrarenal aorta, have a high predictive value for the critical procedure time during standard EVAR under IFU. However, the importance of complex iliac anatomy cannot be overestimated. Beckerman et al. claimed that iliac morphology is the main cause (41.6%) of the lack of fulfilment of the criteria for IFUs in off-label EVAR procedures [24]. In our cohort, the complex anatomy of the iliac arteries, especially the left common iliac artery, was found to be associated with prolonged surgery. This complication was observed in 88.9% of interventions, resulting in prolonged procedure times due to significant challenges with wire navigation, transbrachial access, or conversion to an open procedure. Finally, a high calcification load in the right femoral artery was accompanied by an increase in the intervention time. This observation agrees with the analysis by Bischoff et al., who posited that a high calcification load in the access vessels was the second most common cause, following the neck configuration, contributing to the exclusion of EVAR [25].

## 5. Conclusions

Horos can be used to plan EVAR in cases of uncomplicated anatomy, wherein it exhibits high inter- and intra-observer reliability. BMI has a significant impact on radiation exposure. Thus, it is possible to predict the prospective intraoperative radiation exposure during standard EVAR based on BMI. Additionally, large and elongated iliac arteries contribute to the X-ray burden. The intervention time is influenced by several factors, including BMI, the maximum aortic diameter, the tortuosity of the entire infrarenal aorta, and the angulation and tortuosity index of the iliac arteries. Thus, BMI and the tortuosity of the entire infrarenal aorta are predictors of the duration of standard EVAR.

## 6. Limitations

The Department of Vascular and Endovascular Surgery strictly adhered to the instructions for use and excluded patients with unfavourable anatomies of the proximal and landing zones, as well as those with difficult access vessels for EVAR. These patients instead qualified for open surgery. Moreover, some patients had to be excluded because of the lack of or inadequate imaging.

## Figures and Tables

**Table 1:** Descriptive statistics of the study cohort (men: *n* = 83, 92.2%; women: *n* = 7, 7.8%).

Parameter	Gender	Mean ± Standard Deviation (SD)	Median (Min.; Max.)	95% Confidence Interval (CI)	*p*
Age (years)	Male	73.8 ± 8.3	84 (57; 90)	(71.9; 75.6)	<0.05
	Female	69.4 ± 6.2	68 (60; 77)	(63.7; 75.1)	
Body mass index (BMI) (kg/m^2^)	Male	27.5 ± 4	26.9 (17.6; 39.2)	(26.6; 28.3)	>0.05
	Female	27.6 ± 6.9	26.6 (19.2; 38.4)	(21.1; 34)	

**Table 2:** Summary of potential procedural factors impacting radiation exposure and operation times. The cut-off point between the noncritical and critical groups was determined by the mean ± SD for the dose area product (44.03 Gycm^2^) and the procedure time (210.7 min).

Parameter	Dose Area Product			Procedure Time		
	Noncritical (*n* = 75)	Critical (*n* = 14)	*p*	Noncritical (*n* = 80)	Critical (*n* = 9)	*p*
Open access inguinal on both sides	55 (73.3%)	14 (100%)	<0.05	60 (75%)	9 (100%)	>0.05
Percutaneous access inguinal on both sides	11 (14.7%)	0 (0%)	>0.05	11 (13.8%)	0 (0%)	>0.05
Open access/percutaneous access contralateral	9 (12.0%)	0 (0%)	>0.05	9 (11.3%)	0 (0%)	>0.05
Additional brachial/axillar vascular access	4 (5.3%)	4 (28.6%)	<0.05	4 (5.0%)	4 (44.4%)	<0.05
Femoral TEA on one side	3 (4.0%)	0 (0%)	>0.05	2 (2.5%)	1 (11.1%)	>0.05
Femoral TEA on both sides	1 (1.3%)	1 (7.1%)	>0.05	1 (1.3%)	1 (11.1%)	>0.05
Angioplasty of access vessels	9 (12.0%)	2 (14.3%)	>0.05	10 (12.5%)	2 (22.2%)	>0.05
Conversion from endovascular aortic repair (EVAR) to open aortic repair (OAR)	2 (2.7%)	0 (0%)	>0.05	0 (0%)	2 (22.2%)	<0.05
Access-related bleeding	1 (1.3%)	0 (0%)	>0.05	0 (0%)	1 (11.1%)	>0.05

**Table 3:** (A) Quantitative aneurysm sizing for EVAR planning. The anatomical references for the single parameters can be found in Figure 1. (B) Qualitative aneurysm sizing for EVAR planning.

(A)				
Parameter	Mean ± SD	Median (Min.; Max.)		95% CI
Neck-sac angulation	35.4 ± 20.3	33 (2; 84)		(31.6; 40.46)
D1	22.8 ± 3.2	23 (17; 32)		(22.2; 23.5)
D2	24 ± 3.6	24 (17; 33)		(23.2; 24.8)
D3	26.4 ± 4.1	27 (17; 34)		(25.3; 27.3)
D4	57.1 ± 12	55 (41; 110)		(55.2; 59.9)
D5	27.2 ± 9.6	25 (15; 68)		(25.2; 29.2)
D6 right	14 ± 3.1	14 (8; 25)		(13.3; 14.6)
D6 left	13.3 ± 2.6	13 (6; 20)		(12.8; 13.8)
D7 right	14.7 ± 4.4	14 (7; 40)		(13.8; 15.6)
D7 left	13.4 ± 2.8	13 (8; 22)		(12.9; 14.0)
D8 right	13.7 ± 3.2	14 (7; 23)		(13.0; 14.3)
D8 left	13.7 ± 3.1	13 (7; 26)		(13.0; 14.3)
D9 right	8.2 ± 1.5	8 (5; 12)		(7.9; 8.5)
D9 left	8.2 ± 1.6	8 (4; 12)		(7.9; 8.5)
D10 right	9.1 ± 1.5	9 (6; 13)		(8.8; 9.4)
D10 left	9.2 ± 1.5	9 (5; 13)		(8.8; 9.5)
D11 right	9.7 ± 1.7	10 (7; 14)		(9.4; 10.1)
D11 left	9.8 ± 1.7	10 (7; 14)		(9.4; 10.1)
L1	38.1 ± 15	36 (13; 83)		(35.0; 41.2)
L2	128.4 ± 15.3	129 (93; 181)		(125.2; 131,6)
L3 right	70 ± 18.1	67 (36; 121)		(66.2; 73.8)
L3 left	75 ± 18.3	72 (41; 142)		(71.1; 78.8)
**(B)**				
**Parameter**			***n***	**%**
Tortuosity of infrarenal aortic aneurysm	1.0–1.04		41	45.6
	1.05–1.09		22	24.4
	≥1.1		27	30.0
Tortuosity of common iliac artery	1.0–1.04	Right	18	20.0
		Left	13	14.4
	1.05–1.09	Right	28	31.1
		Left	33	36.7
	≥1.1	Right	44	48.9
		Left	44	48.9
Angulation of common iliac artery	<45°	Right	31	34.4
		Left	37	41.1
	45–90°	Right	40	44.4
		Left	23	25.6
	>90°	Right	14	15.6
		Left	18	20.0
	2 × 90°	Right	5	5.6
		Left	12	13.3
Neck configuration	Straight		35	38.9
	Tapered		1	1.1
	Angulated		44	48.9
	Reverse tapered		3	3.3
	Bulging		6	6.7
	Short		1	1.1

**Table 4:** Intra- and inter-observer variability in all measurements by both observers.

Parameter	Mean ± SD	Median (Min.; Max.)	95% CI
Intra-observer reliability for the first observer	0.86 ± 0.12	0.91 (0.4; 0.97)	(0.81; 0.91)
Intra-observer reliability for the second observer	0.88 ± 0.07	0.89 (0.71; 0.98)	(0.86; 0.91)
Inter-observer reliability for the first sizing round	0.88 ± 0.08	0.89 (0.61; 0.98)	(0.85; 0.91)
Inter-observer reliability for the second sizing round	0.88 ± 0.09	0.9 (0.46; 0.96)	(0.84; 0.91)

**Table 5:** Descriptive statistics for intraoperative results during EVAR.

Parameter	Mean ± SD	Median (Min.; Max.)	95% CI
Procedure time (min)	147.6 ± 71.6	124.5 (60; 465)	(132.6; 162.6)
Dose area product (Gycm^2^)	23 ± 21	14.2 (2.2; 92.8)	(18.5; 27.4)
Radiation time (s)	1699.7 ± 1193.2	1347 (448; 7927)	(1446.9; 1952.5)
Amount of contrast medium (mL)	89.4 ± 57.1	75 (20; 400)	(76.8; 101.9)

**Table 6:** Spearman’s rank correlation analysis of patient-specific, peri-interventional, anatomical parameters, dose area product, and procedure time (ρ—Spearman’s rank correlation coefficient; *p*—significance level).

Parameter	Dose Area Product		Procedure Time	
	ρ	*p*	ρ	*p*
BMI	0.37	<0.01	0.1	>0.05
Radiation time	0.48	<0.001	0.69	<0.001
Amount of contrast medium	0.5	<0.001	0.25	<0.05
Type of endograft	−0.3	<0.01	0.06	>0.05
Neck-sac angulation	0.1	>0.05	0.18	<0.05
Neck diameter	−0.11	>0.05	−0.05	>0.05
Maximum aneurysm diameter	0.18	>0.05	0.3	<0.01
Common iliac artery diameter right	0.24	<0.05	0.16	>0.05
Common iliac artery diameter left	0.06	>0.05	0.1	>0.05
Minimum external iliac artery diameter right	0.14	>0.05	−0.01	>0.05
Minimum external iliac artery diameter left	0.11	>0.05	0.1	>0.05
Common femoral artery diameter right	0.25	<0.05	0.12	>0.05
Common femoral artery diameter left	0.23	<0.05	0.21	>0.05
Tortuosity of infrarenal aortic aneurysm	0.08	>0.05	0.24	<0.05
Tortuosity of common iliac artery right	0.02	>0.05	0.21	<0.05
Tortuosity of common iliac artery left	0.15	>0.05	0.38	<0.001
Angulation of common iliac artery right	0.07	>0.05	0.33	<0.01
Angulation of common iliac artery left	0.25	<0.05	0.5	<0.001

**Table 7:** Univariate analysis of the impact of procedural, patient-specific, and anatomical factors on radiation exposure (dose area product) in logistic regression.

Parameter	Odds Ratio (OR)	95% CI	*p*
BMI	1.177	(1.032; 1.354)	<0.05
Radiation time	1.002	(1.001; 1.003)	<0.05
Amount of contrast medium	1.007	(0.998; 1.017)	>0.05
Diameter of common iliac artery right	0.996	(0.852; 1.121)	>0.05
Diameter of common femoral artery right	1.182	(0.845; 1.668)	>0.05
Diameter of common femoral artery left	1.014	(0.709; 1.424)	>0.05

**Table 8:** Univariate analysis of the impact of procedural, patient-specific, and anatomical factors on procedure time in logistic regression.

Parameter	OR	95% CI	*p*
BMI	1.183	(1.015; 1.389)	<0.05
Radiation time	1.002	(1.001; 1.003)	<0.05
Amount of contrast medium	1.007	(0.996; 1.017)	>0.05
Neck-sac angulation	1.051	(1.016; 1.095)	<0.05
Tortuosity of infrarenal aortic aneurysm	1.124	(1.041; 1.230)	<0.05
Maximum aneurysm diameter	1.054	(1.007; 1.109)	<0.05
Tortuosity of common iliac artery right	1.004	(0.951; 1.045)	>0.05
Tortuosity of common iliac artery left	1.028	(1.002; 1.054)	<0.05

## Data Availability

We do not have permission from the ethics committee to share the datasets due to privacy and ethical restrictions.
